# MDM2 is a novel E3 ligase for HIV-1 Vif

**DOI:** 10.1186/1742-4690-6-1

**Published:** 2009-01-07

**Authors:** Taisuke Izumi, Akifumi Takaori-Kondo, Kotaro Shirakawa, Hiroaki Higashitsuji, Katsuhiko Itoh, Katsuhiro Io, Masashi Matsui, Kazuhiro Iwai, Hiroshi Kondoh, Toshihiro Sato, Mitsunori Tomonaga, Satoru Ikeda, Hirofumi Akari, Yoshio Koyanagi, Jun Fujita, Takashi Uchiyama

**Affiliations:** 1 Department of Hematology and Oncology, Graduate School of Medicine, Kyoto University, 54 Shogoin-Kawaracho, Sakyo-ku, Kyoto 606-8507, Japan; 2 Japanese Foundation for AIDS Prevention, 1-3-12 Misaki-cho, Chiyoda-ku, Tokyo 101-0061, Japan; 3 Department of Clinical Molecular Biology, Graduate School of Medicine, Kyoto University, 54 Shogoin-Kawaracho, Sakyo-ku, Kyoto 606-8507, Japan; 4 Department of Molecular Cell Biology, Graduate School of Medicine, Osaka City University, 1-4-3 Asahi-machi, Abeno-ku, Osaka 545-8585, Japan; 5 CREST, Japan Science Technology Corporation, Kawaguchi, Saitama 332-0012, Japan; 6 Department of Geriatric Medicine, Graduate School of Medicine, Kyoto University, 54 Shogoin-Kawaracho, Sakyo-ku, Kyoto 606-8507, Japan; 7 Central Pharmaceutical Research Institute, Japan Tobacco Inc., 1-1 Murasaki-cho, Takatsuki, Osaka 569-1125, Japan; 8 Laboratory of Disease Control, Tukuba Primate Research Center, National Institute of Biomedical Innovation, Hachimandai-1, Tsukuba, Ibaraki 305-0843, Japan; 9 Laboratory of Viral Pathgenesis, Institute for Virus Research, Kyoto University, 53 Shogoin-Kawaracho, Sakyo-ku, Kyoto 606-8507, Japan

## Abstract

The human immunodeficiency virus type 1 (HIV-1) Vif plays a crucial role in the viral life cycle by antagonizing a host restriction factor APOBEC3G (A3G). Vif interacts with A3G and induces its polyubiquitination and subsequent degradation via the formation of active ubiquitin ligase (E3) complex with Cullin5-ElonginB/C. Although Vif itself is also ubiquitinated and degraded rapidly in infected cells, precise roles and mechanisms of Vif ubiquitination are largely unknown. Here we report that MDM2, known as an E3 ligase for p53, is a novel E3 ligase for Vif and induces polyubiquitination and degradation of Vif. We also show the mechanisms by which MDM2 only targets Vif, but not A3G that binds to Vif. MDM2 reduces cellular Vif levels and reversely increases A3G levels, because the interaction between MDM2 and Vif precludes A3G from binding to Vif. Furthermore, we demonstrate that MDM2 negatively regulates HIV-1 replication in non-permissive target cells through Vif degradation. These data suggest that MDM2 is a regulator of HIV-1 replication and might be a novel therapeutic target for anti-HIV-1 drug.

## Background

Host restriction factors protect hosts from viruses, whereas viruses evade these proteins to replicate more efficiently in host cells. The interplay between the host restriction factors and viral proteins is therefore very important for regulating viral replication [1,2]. A3G (Apolipoprotein B mRNA editing enzyme, catalytic polypeptide-like 3G) is a newly identified anti-HIV-1 host factor [3], which belongs to the APOBEC superfamily of cytidine deaminases, consisting of APOBEC1, APOBEC2, AID (activation-induced cytidine deaminase), APOBEC3(A-H), and APOBEC4 [4]. A3G is incorporated into HIV-1 virions and inhibits HIV-1 replication by inducing G-to-A hypermutation in viral cDNA during reverse transcription [5-8]. HIV-1 Vif counteracts A3G by targeting it for proteasomal degradation, thus supporting HIV-1 replication in non-permissive target cells [9-11]. Vif forms a ubiquitin ligase (E3) complex with Cullin5 (Cul5), Elongin B, and Elongin C and functions as a substrate recognition subunit of this complex to induce ubiquitination and subsequent degradation of A3G [12,13]. Vif also counteracts several APOBEC3 proteins including APOBEC3F (A3F) [14,15]. These observations reconcile the long-standing mystery of why Vif function is necessary for HIV-1 to infect non-permissive cells. On the other hand, it has been shown that intracellular levels of Vif are maintained relatively low by ubiquitination in virus-producing cells [16-18]. Although several groups have reported E3 ligases important for Vif ubiquitination [17,18], the precise roles and mechanisms of Vif ubiquitination remain unclear. Here we demonstrate that MDM2 is a novel E3 ligase for Vif and that it induces ubiquitination and degradation of Vif, thereby regulating HIV-1 replication.

## Results

### MDM2 downregulates cellular Vif levels by inducing its degradation in a proteasome-dependent manner

**Figure 1:**
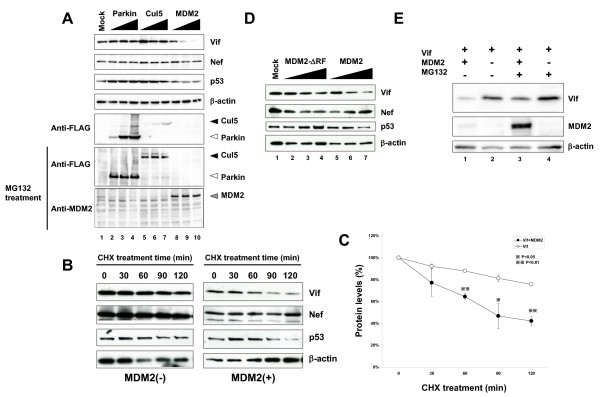
**MDM2 downregulated cellular Vif levels in a proteasome dependent manner**. (A) MDM2 reduced cellular levels of Vif as well as p53, but not that of Nef. HEK293T cells were cotransfected with expression vectors for the indicated E3 ligases and a subgenomic HIV-1 expression vector pNL-A1. Cell lysates were subjected to immunoblotting with the indicated Abs. We could not detect the expression of FLAG-MDM2 without MG132 treatment, because of a rapid degradation of MDM2. MG132 treatment enabled us to detect expression of MDM2 only with anti-MDM2 Ab, but not with anti-FLAG mAb. (B) Twenty-two hours after transfection, the cells were treated with cycloheximide (CHX)(80 μg/ml) for the indicated times, and cell lysates were subjected to immunoblotting with the indicated Abs. (C) The amounts of Vif and Nef were quantified by densitometry, and Vif protein levels were calculated using Nef protein levels as normalizing loading controls and presented as percentage values relative to that without CHX treatment set as 100%. Values are presented as averages of three independent experiments. (D) MDM2 downregulated Vif, but a ΔRF mutant did not. HEK293T cells were cotransfected with expression vectors for MDM2 and the mutant together with pNL-A1, and cell lysates were subjected to immunoblotting with the indicated Abs. (E) p53^-/-^MDM2^-/- ^DKO-MEF cells were cotransfected with expression vectors for MDM2 and Vif, and treated with 10 μM MG132 for 6 hrs, and cell lysates were subjected to immunoblotting with the indicated Abs.

To investigate the biological roles and molecular mechanisms of Vif ubiquitination, we tried to identify a novel E3 ligase that may be involved in the ubiquitination of Vif. During a search for Vif-interacting proteins in the HIV, Human Protein Interaction Database of National Institute for Allergy & Infectious Diseases , we were struck by a protein called Gankyrin (proteasome 26S subunit, non-ATPase, 10 (PSMD10)). We first examined the biological effects of Gankyrin, but could not detect a downregulation of Vif (data not shown). As we previously reported that Gankyrin itself doesn't have an enzymatic activity and that it rather enhances the E3 ligase activity of MDM2 on p53 ubiquitination and degradation as a co-factor [19], we tested the possibility that MDM2 plays an important role in Vif ubiquitination as a novel E3 ligase. We examined the effect of several E3 ligases including MDM2 (a RING finger type E3 that mediates p53 ubiquitination and degradation [20]), Cul5 (another RING finger type E3 that forms a complex with Vif and is reported to induce Vif ubiquitination [17,21]), and Parkin (another RING finger type E3) on cellular Vif levels (Fig. 1A). HEK293T cells were transfected with a subgenomic expression vector pNL-A1 that expressed all HIV-1 proteins except for *gag *and *pol *products [22], together with the expression plasmids for these E3 ligases. We found that the ectopic expression of MDM2 downregulated the cellular levels of Vif as well as p53 in transfected cells in a dose-dependent manner (Fig. 1A, lanes 8–10), whereas Parkin and Cul5 did not affect their cellular levels (lanes 2–4 and 5–7, respectively), even though the latter proteins were expressed more than MDM2. Our results are discrepant with previous reports that demonstrated Cul5 induced Vif ubiquitination and degradation [17,23]. We assume that overexpression of Cul5 alone is insufficient to induce Vif degradation, because other E3 components are not overexpressed. Ectopic expression of MDM2 did not affect cellular levels of another viral protein such as Nef, suggesting that MDM2 specifically downregulated Vif levels; this result also excluded the possibility that MDM2 affected the transcriptional activity of the HIV-1 LTR.

Because it is well known that MDM2 regulates p53 levels by modulating its protein stability, we next examined the protein stability of Vif with the ectopic expression of MDM2. HEK293T cells were transfected with pNL-A1 with or without a MDM2 expression vector and treated with cycloheximide 21 hrs after transfection. After cycloheximide treatment, cellular levels of Vif decreased by 60% in MDM2-transfected cells and by 20% in control cells, respectively (Fig. 1B &1C), indicating that Vif decayed much faster when MDM2 was overexpressed. The stability profile of Vif protein was similar to that of p53 (Fig. 1B). However, in our hands, the half-life of Vif protein was longer than those shown in previous studies from several laboratories. We interpret that this difference is attributable to divergent methods used in the studies which employed radioisotopes or cycloheximide. Thus, our findings suggest that MDM2 affects the stability of Vif protein similar to its effect on p53. We also examined the stability of Vif in MDM2-/- MEF cells. Vif decayed much faster in p53-/- MEF cells than in p53-/-MDM2-/- double knock-out (DKO) MEF cells (Additional file 1), suggesting that endogenous MDM2 can also influence the stability of Vif. We then tested a RING finger domain-deleted MDM2 mutant, ΔRF, which is inactive for the ubiquitination activity of MDM2 [24]. Ectopic expression of MDM2 suppressed cellular Vif levels, but the expression of ΔRF did not (Fig. 1D). This result suggests that ubiquitination of Vif by MDM2 is involved in the downregulation of cellular Vif levels. We further treated transfected cells with a proteasome inhibitor MG132 to see whether the downregulation of Vif by MDM2 was proteasome-dependent. Treatment with MG132 clearly restored the cellular Vif level that was downregulated by MDM2 (Fig. 1E, top panel, lane 3 as compared with lane 1), supporting that the MDM2-mediated downregulation of Vif was proteasome-dependent. Taken together, we concluded that MDM2 downregulates cellular Vif level by inducing its degradation in a proteasome-dependent manner.

### MDM2 specifically binds and downregulates Vif

**Figure 2:**
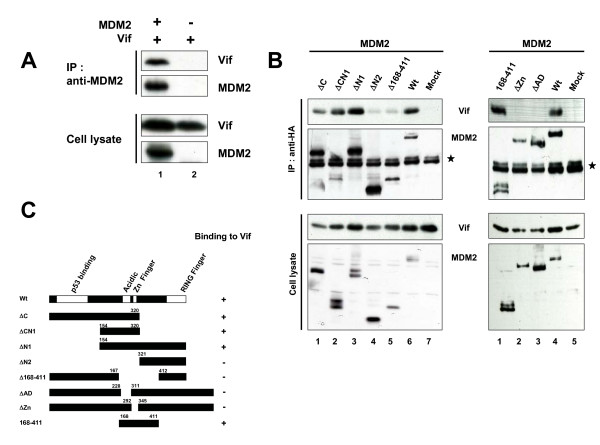
**MDM2 bound Vif in its central domain**. (A) Immunoprecipitation assays revealed the interaction of MDM2 with Vif *in vivo*. HEK293T cells were cotransfected with expression vectors for MDM2 and Vif and treated with MG132 for 6 hrs prior to harvest. Cell lysates were immunoprecipitated with anti-MDM2 mAb followed by immunoblotting with the indicated Abs (upper two panels). Cell lysates were also subjected to immunoblotting with the indicated Abs (lower two panels). (B) The interaction domain of MDM2 with Vif. HEK293T cells were cotransfected with expression vectors for HA-tagged MDM2 wild type (Wt) and mutants together with pNL-A1, and cell lysates were immunoprecipitated with anti-HA mAb followed by immunoblotting with the indicated Abs. Asterisk indicates immunoglobulin heavy chains from thenimmunoprecipitation. (C) Schematics of MDM2 mutants binding to Vif are shown.

**Figure 3:**
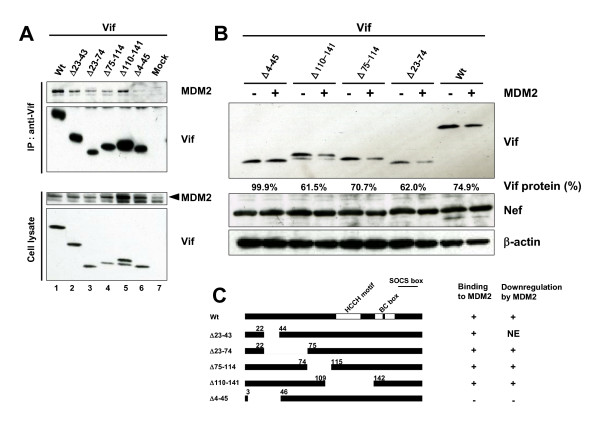
**MDM2 specifically bound and downregulated Vif**. (A) The interaction domain of Vif with MDM2. HEK293T cells were cotransfected with expression vectors for Vif and mutants together with pCMV/HA-MDM2, and cell lysates were immunoprecipitated with anti-Vif mAb followed by immunoblotting with the indicated Abs. Arrowhead indicates MDM2. (B) The downregulation of Vif protein by MDM2. HEK293T cells were cotransfected with expression vectors for Vif and mutants with or without pCMV/HA-MDM2, and cell lysates were subjected to immunoblotting with the indicated Abs. The amounts of Vif were quantified by densitometry and shown as the protein ratio relative to that without expression of MDM2. (C) Schematics of Vif mutants bound by and downregulated by MDM2. NE: not examined.

To further investigate the molecular link between MDM2 and Vif, we next examined the physical interaction of MDM2 with Vif. Immunoprecipitation assays showed that Vif was co-precipitated with MDM2 (Fig. 2A). Glutathione S-transferase (GST) pull-down assays showed that MDM2 was found in GST-Vif-bound, but not GST-bound, material (data not shown). Using a series of MDM2 deletion mutants, we determined that the central region of MDM2 (amino acids 168–320) was necessary for Vif binding (Fig. 2B, left panel &2C). To more precisely determine a Vif-binding domain, we further tested mutants deleted in a Zn Finger domain (ΔZn) or in an acidic domain (ΔAD). Neither mutant could bind Vif, whereas the mutant containing amino acids 168–411 was able to bind Vif, suggesting that both domains are necessary and that the central domain is sufficient for Vif binding (Fig. 2B, right panel &2C). Additionally, using a series of Vif deletion mutants, we also found that the N-terminal region of Vif (amino acids 4–22) is needed for MDM2 binding (Fig. 3A &3C). Furthermore, we examined the MDM2-mediated downregulation of Vif mutants. MDM2 was able to efficiently downregulate cellular levels of the MDM2-binding Vif mutants but not that of an MDM2-non binding mutant, Δ4–45 (Fig. 3B). Collectively, these results indicated that the Vif-MDM2 interaction is required for MDM2-mediated downregulation of Vif (Fig. 3C).

### MDM2 induces ubiqutination of Vif

**Figure 4:**
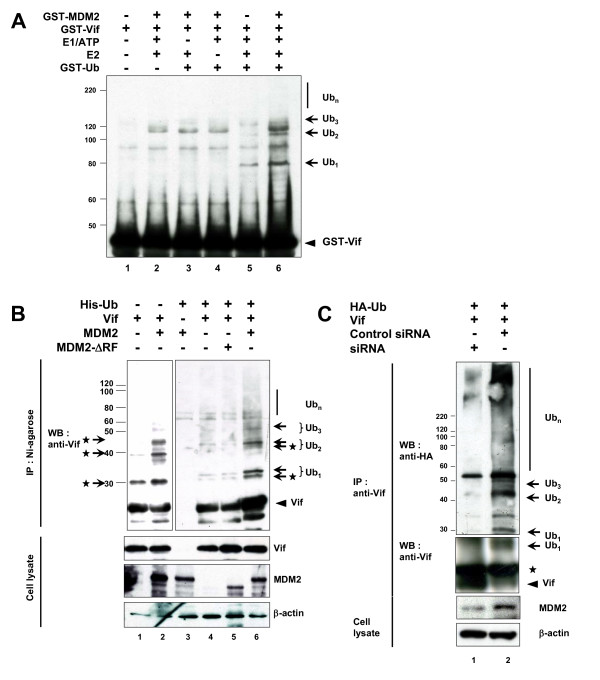
**MDM2 induced the polyubiquitination of Vif *in vitro *and *in vivo***. (A) GST-MDM2 induced the polyubiquitination of Vif *in vitro*. Bacterially expressed GST-Vif was subjected to *in vitro *ubiquitination assays. The reaction was performed in the presence or absence of E1, E2, GST-MDM2, and GST-Ubiquitin as indicated. Reactions were subjected to immunoblotting with anti-Vif mAb. Arrows indicate GST-ubiquitin-conjugated Vif. (B) Overexpressed MDM2 induced the polyubiquitination of Vif *in vivo*. HEK293T cells were cotransfected with expression vectors for MDM2 Wt and a ΔRF mutant together with expression vectors for Vif and His-Ubiquitin (His-Ub) as indicated. Cells were treated with MG132 for 6 hrs, and cell lysates were precipitated with Ni-NTA agarose beads followed by immunoblotting with the indicated Abs. Since Vif naturally bound to Ni-NTA agarose, we detected a Vif band itself (arrowhead), whereas no signal was detected in cells lacking Vif (lane 3). Arrows indicate His-Ub-conjugated Vif. Arrows with asterisk indicate Vif conjugated with endogenous ubiquitin. (C) Transduction of siRNA reduced cellular levels of endogenous MDM2 and polyubiquitination of Vif. HEK293T cells were cotransfected with expression vectors for MDM2 siRNA and control siRNA together with expression vectors for Vif and HA-Ubiquitin (HA-Ub). Cell lysates were immunoprecipitated with anti-Vif mAb followed by immunoblotting with the indicated Abs. Asterisk indicates immunoglobulin light chains from the immunoprecipitation.

Since we found that MDM2 bound Vif and promoted its degradation via a proteasomal pathway, we next examined whether MDM2 is involved in the polyubiquitination of Vif. *In vitro *ubiquitination assays revealed that bacterially expressed GST-MDM2 was able to induce the polyubiquitination of purified GST-Vif protein *in vitro *(Fig. 4A). The ubiquitination of Vif by MDM2 was specific, as the omission of ubiqutin, E1, E2, or MDM2 prevented Vif-ubiquitination as shown in our previous experiments [13]. We also performed *in vitro *ubiquitination assays using immunopurified MDM2 and Cul5. Immunopurified MDM2 was able to induce ubiquitination of Vif *in vitro *to the same extent as Cul5 (Additional file 2, part A), while it could not ubiquitinate the N-terminal Vif deletion mutant Δ22 that was defective for binding MDM2 (Additional file 2, part B). These findings suggest that the interaction with MDM2 is important for Vif ubiquitination. We performed *in vivo *ubiquitination assays to further investigate the importance of MDM2 in Vif ubiquitination. Lysates of cells co-expressing Vif, either with an MDM2 wild type (Wt) or a ΔRF mutant, and His-tagged Ubiquitin (His-Ub) were analyzed for the presence of ubiquitinated Vif conjugates (Fig. 4B). Unfortunately, we detected a Vif band that non-specifically bound to Ni-NTA agarose (arrowhead) due to its nature as a sticky protein. Overexpression of MDM2 induced a ladder detected by anti-Vif Ab, even in the absence of His-Ub (lane 2), suggesting that this ladder represented Vif protein polyubiquitinated with endogenous Ub (arrows with asterisk). Furthermore, in the presence of His-Ub, we detected a doublet of ladder which presumably represented Vif protein polyubiquitinated with endogenous and His-tagged Ub (arrows with asterisk and arrows, respectively). We also obtained similar results using a UbiQapture™-Q Kit (data not shown). We thus concluded that the overexpression of exogenous MDM2 efficiently induced polyubiquitination of Vif *in vivo*. Furthermore, the knock-down of endogenous MDM2 expression by introduction of MDM2-specific short interfering RNA (siRNA) resulted in a significant reduction in the amount of polyubiquitinated Vif, commensurate with the extent of reduced MDM2 expression (Fig. 4C). Collectively, these data indicated that MDM2 mediates polyubiquitination of Vif both *in vitro *and *in vivo*.

### MDM2 negatively regulates HIV-1 replication in non-permissive cells through ubiqutination and degradation of Vif

**Figure 5:**
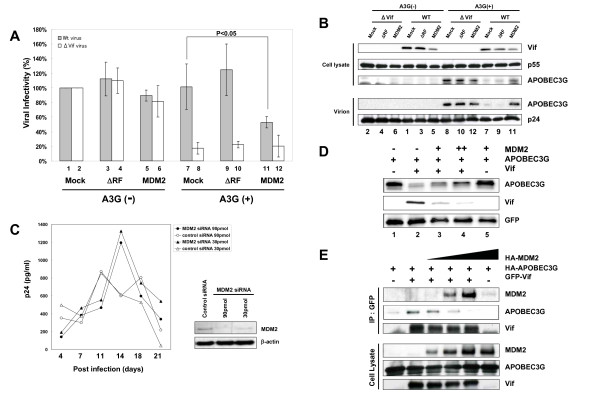
**MDM2 negatively regulated HIV-1 replication in non-permissive cells through the degradation of Vif**. (A) The overexpression of MDM2 inhibited HIV-1 replication in the presence of A3G. NL-43 Wt and ΔVif viruses were produced from HEK293T cells transfected with expression vectors for MDM2 Wt and a ΔRF mutant in the presence or absence of A3G. The viral infectivity was examined using M8166 cells. Values are presented as averages of more than 3 independent experiments. (B) MDM2 reduced cellular levels of Vif, resulting in more incorporation of A3G into HIV-1 virions. Immunoblotting for cell lysates (upper 3 panels) and precipitated virions (lower 2 panels) was performed with the indicated Abs. Lane numbers correspond to those in Fig. 4A. (C) HIV-1 replication in macrophages transfected with MDM2- and control-siRNA. MDM were transfected with MDM2- and control-siRNA and challenged with R5 HIV-1_JR-FL _(left panel). Cell lysates were subjected to immunoblotting with the indicated antibodies (right panels). (D) Coexpression of MDM2 reduced cellular levels of Vif and inversely increased A3G levels in a dose dependent manner. HEK293T cells were cotransfected with expression vectors for A3G, Vif, GFP, and MDM2 as indicated. Cell lysates were subjected to immunoblotting with the indicated Abs. (E) Immunoprecipitation assays revealed that the coexpression of MDM2 blocked the binding of A3G to Vif in a dose dependent manner. HEK293T cells were cotransfected with expression vectors for A3G, GFP-Vif, and MDM2 as indicated. Cell lysates were immunoprecipitated with anti-GFP mAb followed by immunoblotting with the indicated Abs.

Next, we examined the effect of MDM2 on HIV-1 replication. In a single round infection assay (Fig. 5A), in the absence of A3G, viral replication was not affected by expression of MDM2 and/or Vif (lanes 1–6). In contrast, in the presence of A3G in a non-permissive cell setting, without the expression of MDM2, the wild type virus could replicate but the ΔVif virus could not, as previously reported (lanes 7 & 8) [3,8]. Co-expression of MDM2 reduced the cellular level of Vif (Fig. 5B, upper panel, lanes 5 & 11), resulting in the increased virion incorporation of A3G (Fig. 5B, 2nd lower panel, lane 11 as compared with lanes 7) and the greater suppression of viral replication (Fig. 5A, lane 11 as compared with lane 7).

We also tested the effect of MDM2 on HIV-1 replication in the presence of A3F. MDM2 suppressed viral replication in the presence of A3F, similar to results shown for A3G (Additional file 3). These data indicated that the MDM2-mediated Vif downregulation led to upregulated cellular A3G and A3F levels in producer cells, resulting in less infectious HIV-1 virions produced. Since MDM2 was previously reported to upregulate HIV-1 transcription by ubiquitination of Tat, we further examined HIV-1 replication in macrophages knocked down for MDM2 (Fig. 5C). We chose terminally differentiated macrophages as the target, because the knockdown of MDM2 is lethal for proliferating cells. HIV-1 replicated more efficiently in macrophages transfected with MDM2 siRNA than in control siRNA-transfected macrophages. These data indicated that MDM2 negatively regulated HIV-1 replication in non-permissive target cells through the ubiquitination and degradation of Vif.

To obtain further insights into the mechanisms why our MDM2 system did not induce the ubiquitination of A3G which was bound to Vif, we tested the expression levels and the binding affinity of A3G to Vif in transfected cells. Co-expression of MDM2 reduced the cellular levels of Vif and inversely increased the A3G levels in a dose dependent manner (Fig. 5D). Immunoprecipitation assays revealed that the co-expression of MDM2 blocked the binding of A3G to Vif in a dose dependent manner (Fig. 5E). These data suggest that the interaction between MDM2 and Vif precludes A3G from binding to Vif.

## Discussion

In this study, we report that MDM2 is a novel E3 ligase for HIV-1 Vif. MDM2 physically interacts with Vif and functions as an E3 ligase for Vif to induce its polyubiquitination and proteasomal degradation. Several E3 ligases including Cul5 [17], Nedd4, and AIP4 [18], have been reported to induce Vif ubiquitination, and the roles of Cul5 for Vif ubiquitination and degradation are especially well documented. Dang et al. have recently reported that Cul5 induces A3G degradation not by direct ubiquination of A3G but indirectly through Vif ubiqutination and that polyubiquitinated Vif might serve as a vehicle to transport A3G into proteasomes for degradation [23]. In this manuscript, we show that MDM2 only targets Vif for degradation but not A3G, although MDM2 and Cul5 both induce Vif ubiquitination (Additional file 2, part A). MDM2 reduced cellular Vif levels and inversely increased A3G levels (Fig. 5B &5D), unlike Cul5. One possible explanation is that the binding of MDM2 to Vif precluded A3G from binding Vif (Fig. 5E), whereas a Cul5-Vif complex can bind A3G to form a ternary complex. MDM2 binds the N-terminal region of Vif which does not overlap with, but is close to the A3G/A3F binding domain [25]. This binding might affect the interaction of Vif with A3G and/or A3F. Furthermore, the evidence that an MDM2 ΔRF mutant failed to protect A3G indicated that the ubiquitination and degradation of Vif is necessary to protect A3G and A3F from Vif. These findings suggest that different E3 ligases might play different roles in Vif ubiquitination. Further studies on the different roles of Vif ubiquitination by different E3 ligases and their virological significance should be investigated.

We demonstrate that MDM2 negatively regulated HIV-1 replication through Vif degradation. Through the degradation of target proteins (p53, pRB, etc), MDM2 can exert profound physiological effects on the regulation of cell cycle, cell proliferation, DNA repairs and other processes. To our knowledge, this is the first report to show that MDM2 plays an important role in viral replication through the degradation of viral proteins. Recently, MDM2 was also reported to ubiquitinate HIV-1 Tat protein and activate its transcriptional activity in a non-proteolytic manner [26]. Our experiment using MDM2 knockdown macrophages showed that HIV-1 replication in these macrophages was more efficient than in control siRNA-transfected macrophages. These data are consistent with MDM2 negatively regulateing HIV-1 replication through Vif ubiquitination (Fig. 5C). However, the replication efficiency of HIV-1 in MDM2 knockdown macrophages was only 2-fold higher and was slower than in control siRNA-transfected macrophages. This suggests the possibilities that the ubiquitination of Tat might work as a positive regulatory factor at an earlier phase of infection and that MDM2 might be involved in both positive and negative regulation of HIV-1 replication at different stages. Further studies on the detailed effect of MDM2 on HIV-1 replication are needed.

We also demonstrated that Vif can bind MDM2 directly. We also mapped the interaction domain of MDM2 with Vif to amino acids 168–320 which is located in its central acidic and Zn finger domains. This central domain is different from the primary p53-binding site of MDM2 which is located in its N-terminal region; however, this central deomain was recently reported as a second p53-binding site and was shown to be important for the regulation of p53 stability [27-30] (Fig. 2B &2C). Interestingly, several proteins including p300, p14^ARF^, and pRB bind to the central domain of MDM2 and regulate the stability and function of p53 via MDM2 [28,31]. Thus, it is possible that Vif might affect the stability and function of p53. Indeed, we confirmed that Vif can stabilize p53 (*Izumi et al., unpublished data*), which could explain why the effect of MDM2 on p53 degradation was weaker than that on Vif as shown in Fig. 1A. A further study is under way to elucidate this new function of Vif (*Izumi et al., HIV-1 Vif induces G2 cell cycle arrest via the p53 pathway, unpublished*).

Finally, expanding evidence suggests that the ubiquitination system plays important roles in many aspects of HIV-1 replication including the degradation of A3G by Vif [9-11], the degradation of CD4 by Vpu [32], HIV-1 viral budding [33], Tat-mediated transactivation [26], and Vpr-induced G2 cell cycle arrest [34,35]. The functional linkage between Vif and MDM2 also suggests that ubiquitin processes such as the A3G/Vif interplay is highly complex. It is obvious that HIV-1 replication in target CD4+ T cells is strongly affected by the interplay of these proteins. From the viral point of view, this interplay might give an advantage to HIV-1 replication. One possibility is that MDM2 regulates cellular Vif levels appropriately, such as not to affect viral replication [36] but just enough to antagonize A3G. Recent studies suggest that the G-to-A mutations induced by A3G may not be the mechanism by which A3G restricts or controls viral replication [37] and that a partially effective Vif inhibitor may actually accelerate the evolution of drug resistance and immune escape [38]. The inhibitory activity of MDM2 toward Vif could be partially effective and therefore could lead to viral evolution of drug resistance and immune escape. More recently, Nathans et al. have reported a small molecule that specifically antagonizes Vif function and inhibits viral replication by targeting the A3G/Vif axis. This compound enhances Vif degradation only in the presence of A3G, but does not induce A3G degradation and rather stabilizes A3G. They suggested the possibility of a new proteolytic enzyme for Vif degradation and that their new compound interferes with Vif interaction with a host protein in a Vif-A3G-host protein complex, thereby making Vif less stable. The precise biological significance of this Vif-A3G-host protein complex requires future elucidation. Nevertheless, modification or intervention of such Vif-A3G-host protein interplay could lead to the development of new therapeutic strategies for HIV-1 infection.

## Conclusion

MDM2 is a novel E3 ligase for Vif which induces the polyubiquitination and degradation of Vif to negatively regulate HIV-1 replication.

## Methods

### Plasmid constructs

Expression vectors for hemagglutinin (HA)- or FLAG-tagged MDM2, pCMV4/HA-MDM2 or pCMV4/FLAG-MDM2, and their mutants were constructed as previously described [19]. An expression vector for HA-tagged human APOBEC3G, pcDNA3/HA-hA3G [39], and HIV-1 reporter plasmids, pNL43/Δenv-Luc (WT) and pNL43/ΔenvΔvif-Luc (ΔVif) [8], were constructed as previously described. Expression vectors for FLAG-tagged Parkin and Cul5 (pcDNA3/FLAG-Parkin and pcDNA3/FLAG-Cul5, respectively) were constructed by the PCR method. Complementary DNA for HIV-1 Vif was also cloned into pDON-AI (TAKARA BIO INC.) and pDON/EGFP for expression of Vif and EGFP-fused Vif (EGFP-Vif). The subgenomic expression vector pNL-A1, which expresses all HIV-1 proteins except for *gag *and *pol *products, and its mutants expressing Vif deletion mutants were kind gifts from Dr. K. Strebel [22].

### Co-immunoprecipitation assays

We performed an immunoprecipitation assay for protein-protein interaction *in vivo*, as described previously [8]. HEK293T cells were cotransfected with pCMV4/HA-MDM2 and pNL-A1 by the calcium phosphate method. Two days after transfection, cells were lysed in lysis buffer (25 mM HEPES pH7.4/150 mM NaCl/1 mM MgCl_2_/0.5% TritonX-100/10% Glycerol) and complexes were immunoprecipitated with anti-MDM2 monoclonal antibody (mAb) (SMP-14, Santa Cruz Biotechnology, Inc., Santa Cruz, CA and Ab-1, Calbiochem, EMD Biosciences, Inc, Darmstadt, Germany) and Protein A-Sepharose beads (Amersham Biosciences Corp.) at 4°C. The beads were washed with RIPA buffer (50 mM Tris-HCl pH8.0/150 mM NaCl/1% Triton-X 100/0.1% SDS/0.1% DOC) and analyzed by immunoblotting with anti-Vif mAb (#319) (A kind gift from Dr. M. Malim through the AIDS Research and Reference Reagent Program) [40] or anti-HA mAb (12CA5). To map the regions of MDM2 necessary for binding to Vif, HEK293T cells were cotransfected with expression vectors for a series of MDM2 deletion mutants together with pNL-A1. Complexes were immunoprecipitated with anti-HA mAb and analyzed by immunoblotting with anti-Vif mAb. To map the regions of Vif necessary for binding to MDM2, HEK293T cells were cotransfected with expression vectors for a series of Vif deletion mutants together with pCMV4/HA-MDM2. Complexes were immunoprecipitated with anti-Vif mAb and analyzed by immunoblotting with anti-MDM2 mAb. In all these experiments, transfected cells were treated with MG132 for 6 hrs prior to harvesting in order to stabilize both Vif and MDM2; otherwise we could not detect the expression of MDM2 because of its rapid degradation, as seen in Fig. 1A.

### In vitro and in vivo ubiquitination assays

*In vitro *ubiquitination assays were carried out in ubiquitin reaction buffer (50 mM Tris-HCl/2 mM ATP/5 mM MgCl_2_/2 μM DTT) with E1(200 ng), E2(Ubc5c)(150 ng), and GST-tagged ubiquitin (GST-Ub) (10 μg) as described previously [13]. MDM2 and Vif were expressed as GST-fusion proteins in Escherichia coli strain DH5α and BL21, respectively. The reactions were incubated at 30°C for 90 min. The samples were subjected to immunoblotting with anti-Vif mAb to detect GST-ubiquitin conjugated Vif.

For *in vivo *ubiquitination assays, HEK 293T cells were cotransfected with plasmids expressing Vif, FLAG-MDM2 or its mutants, and His-tagged ubiquitin (His-Ub) as indicated. Cells were treated with 10 μM MG132 for 6 hrs prior to harvesting. Forty-eight hours post transfection, cell lysates were affinity-purified with Ni-NTA-agarose beads (Invitrogen corporation, Carlsbad, CA) and analyzed by immunoblotting with anti-Vif mAb.

For production of RNAi within the cells, we used the pSuper vector as described previously [19]. pSuper-MDM2-1 contained the 19 nt derived from the *mdm2 *cDNA (nt 404–422) as the target sequence. Double-stranded RNA containing scrambled 19 nt was used as a control. HEK293T cells were transfected with pSuper plasmids together with plasmids expressing Vif and HA-Ub. Cell lysates were immunoprecipitated with anti-Vif mAb followed by immunoblottimg with anti-HA mAb.

### Single round infection assays with HIV-1 luciferase reporter virus

Luciferase reporter viruses with or without Vif were prepared by cotransfection of pNL43/Δenv-Luc (Wt) or pNL43/ΔenvΔvif-Luc (ΔVif) plus pVSV-G together with a mock vector or an expression vector for MDM2 or a mutant in the presence or absence of pcDNA3/hA3G by calcium phosphate as previously described [8]. The reporter viruses were adjusted according to p24 values and used to infect M8166 target cells. Productive infection was measured by luciferase activity and values were presented as percent infectivity relative to the value of each virus without the expression of hA3G.

### Knockdown of MDM2 in macrophages and replication assays

Monocyte-derived macrophages (MDM) were cultured for 7 days from CD14+ monocytes isolated from the peripheral blood of an HIV-1-negative healthy individual. Electroporation with Stealth Select RNAi for MDM2 or Control (Invitrogen Corporation) was performed using the Nucleofector machine (Amaxa Inc., Gaithersburg, MD) according to the manufacturer's instructions. Twenty four hours after transfection, MDM were challenged with R5 HIV-1_JR-FL _at multiplicity of infection of 0.1 at 37°C for 3 hrs. The cells were cultured from day 4 to 21 after infection, and the concentration of p24 antigen in the supernatant was measured with an HIV-1 p24 antigen enzyme-linked immunosorbent assay [ELISA] kit (ZeptMetrix, Buffalo, NY).

## Competing interests

The authors declare that they have no competing interests.

## Authors' contributions

TI. designed research, performed research, contributed vital new reagents, analyzed data, and wrote the paper. ATK designed research, analyzed data, wrote the paper, and organized the research. KS, KIo, and MM prepared the materials and performed a part of the research. KIwai, HK, TS, MT, SI., and HA contributed vital new reagents. YK contributed vital new reagents, performed a part of the research, and analyzed the data. HH, KItoh, and JF designed the research, contributed vital new reagents, and analyzed the data. TU analyzed the data, drafted the paper, and organized the research.

## Supplementary Material

Additional file 1 **Supplementary figure 1 – the stability of Vif protein in p53-/- MEF and p53-/-MDM2-/- MEF cells.** MEF cells were transfected with pDON/Vif or pcDNA3/HA-A3G. Twenty-two hours after transfection, the cells were treated with cycloheximide (CHX) for the indicated times, and cell lysates were subjected to immunoblotting with the indicated Abs. Click here for file

Additional file 2 **Supplementary figure 2 – immunopurified MDM2 induced the polyubiquitination of Vif *in vitro*.** (A) MDM2 as well as Cul5 induced the polyubiquitination of Vif. HEK293T cells were transfected with expression vectors for His-MDM2 and His-Cul5. His-tagged proteins were purified using Ni-NTA agarose and subjected to *in vitro *ubiquitination assays as described in a legend to Fig. 4A. Reactions were subjected to immunoblotting with anti-Vif Ab. Arrows indicate GST-Ub-conjugated Vif. Asterisks indicate non-specific bands associated with GST-Vif protein recognized by anti-Vif Ab, as they are seen in lanes 1 and 3. (B) MDM2 induced the polyubiquitination of Vif Wt but not that of Δ22 that was defective for binding MDM2. Filled asterisks indicate non-specific bands associated with GST-Vif protein, while white asterisks indicate those associated with GST-Vif Δ22. Click here for file

Additional file 3 **Supplementary figure 3 – the overexpression of MDM2 inhibited HIV-1 replication in the presence of A3F.** Single round infection assays were performed in the presence or absence of A3F as described in a legend to Fig. 5A. Values are presented as averages of more than 3 independent experiments. Click here for file
