# Supplementary figures and images for: MDM2 is a novel E3 ligase for HIV-1 Vif
Source: Retrovirology. 2009 Jan 7;6:1. doi: 10.1186/1742-4690-6-1 (PMC2629459; doi:10.1186/1742-4690-6-1)

**CHX treatment time (min)**

**0 30 60 90 120 150**

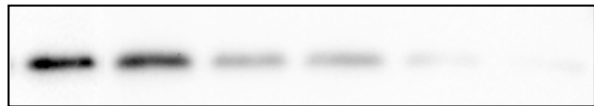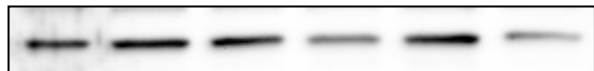

**p53-/- MEF**

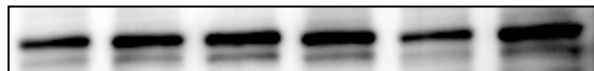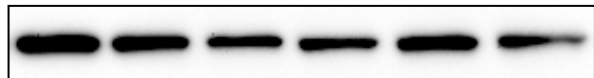

**CHX treatment time (min)**

**0 30 60 90 120 150**

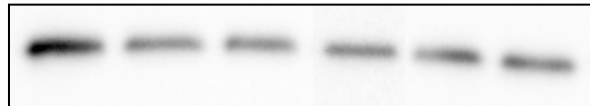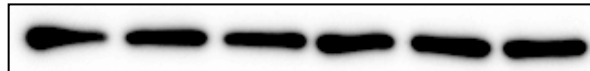

**p53-/-MDM2-/- MEF**

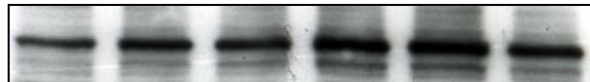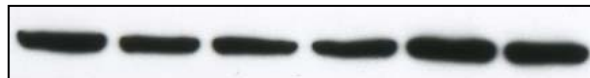

**Vif**

**β-actin**

**APOBEC3G**

**β-actin**

Supplement: Additional file 1 — Supplementary figure 1 – the stability of Vif protein in p53-/- MEF and p53-/-MDM2-/- MEF cells. MEF cells were transfected with pDON/Vif or pcDNA3/HA-A3G. Twenty-two hours after transfection, the cells were treated with cycloheximide (CHX) for the indicated times, and cell lysates were subjected to immunoblotting with the indicated Abs. [file 1742-4690-6-1-S1.pdf]

**A**

|          |   |   |   |   |
|----------|---|---|---|---|
| His-Cul5 | - | + | - | - |
| His-MDM2 | - | - | - | + |
| GST-Vif  | + | + | + | + |
| E1/ATP   | + | + | + | + |
| E2       | + | + | + | + |
| GST-Ub   | + | + | + | + |

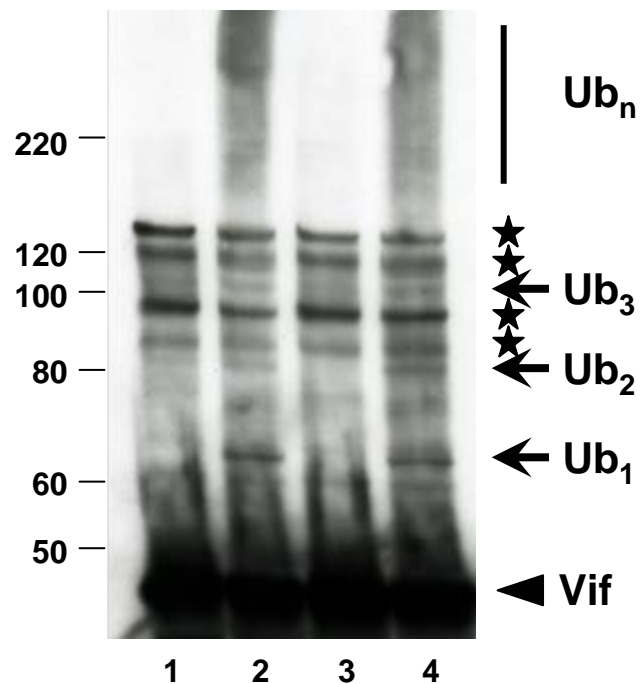**B**

|                     |   |   |   |   |
|---------------------|---|---|---|---|
| His-MDM2            | - | + | - | + |
| GST-Vif $\Delta$ 22 | + | + | - | - |
| GST-Vif             | - | - | + | + |
| E1/ATP              | + | + | + | + |
| E2                  | + | + | + | + |
| GST-Ub              | + | + | + | + |

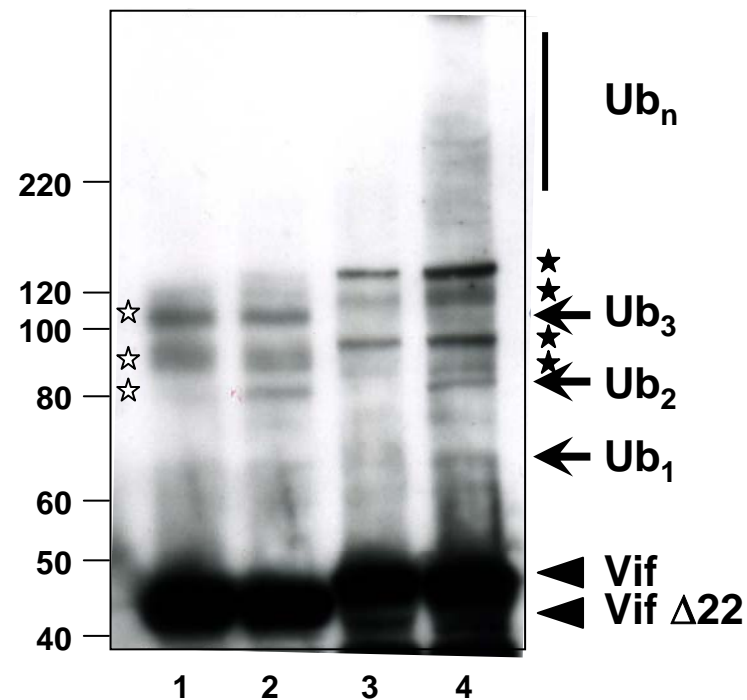

Supplement: Additional file 2 — Supplementary figure 2 – immunopurified MDM2 induced the polyubiquitination of Vif in vitro. (A) MDM2 as well as Cul5 induced the polyubiquitination of Vif. HEK293T cells were transfected with expression vectors for His-MDM2 and His-Cul5. His-tagged proteins were purified using Ni-NTA agarose and subjected to in vitro ubiquitination assays as described in a legend to Fig. 4A. Reactions were subjected to immunoblotting with anti-Vif Ab. Arrows indicate GST-Ub-conjugated Vif. Asterisks indicate non-specific bands associated with GST-Vif protein recognized by anti-Vif Ab, as they are seen in lanes 1 and 3. (B) MDM2 induced the polyubiquitination of Vif Wt but not that of Δ22 that was defective for binding MDM2. Filled asterisks indicate non-specific bands associated with GST-Vif protein, while white asterisks indicate those associated with GST-Vif Δ22. [file 1742-4690-6-1-S2.pdf]

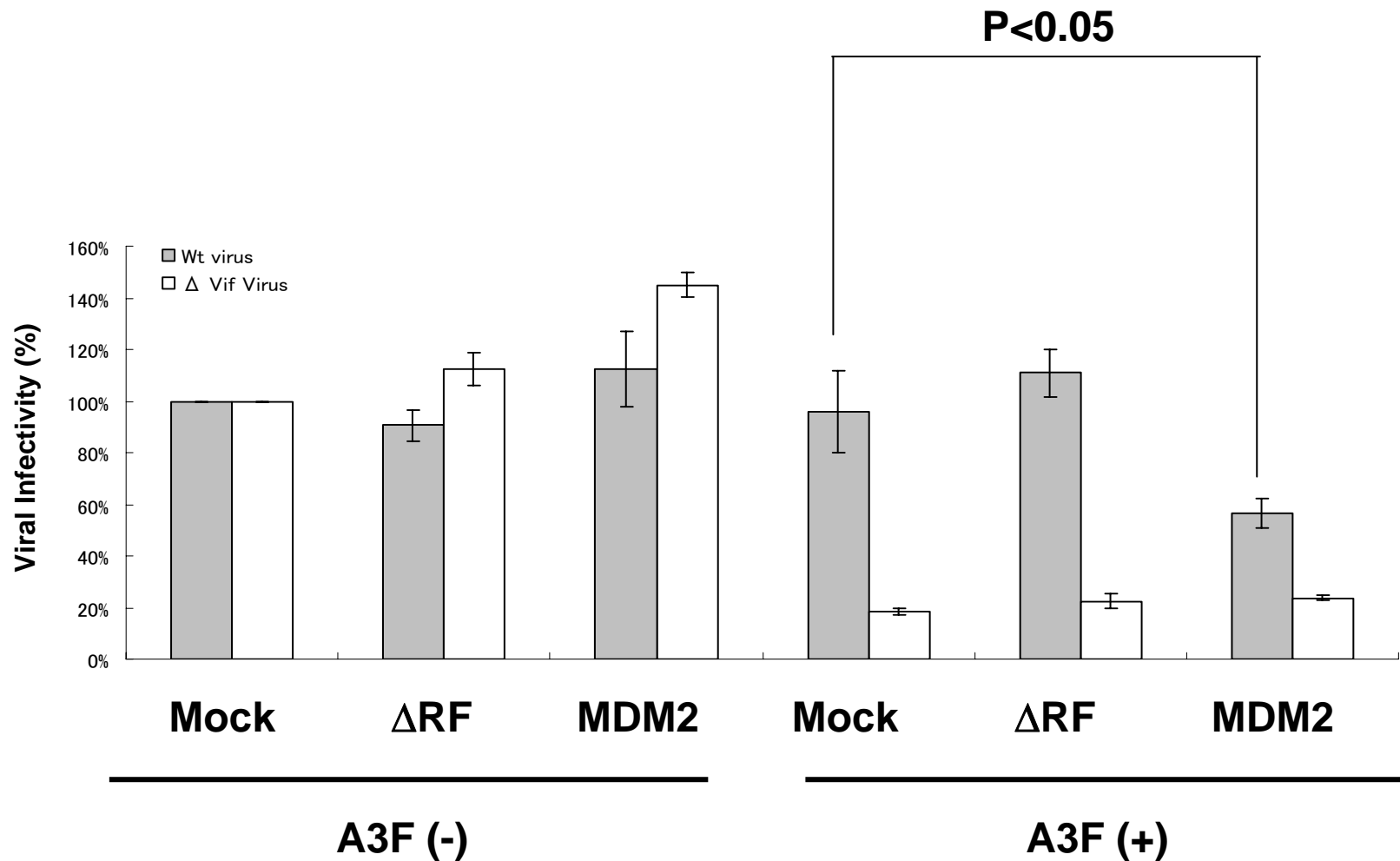

Supplement: Additional file 3 — Supplementary figure 3 – the overexpression of MDM2 inhibited HIV-1 replication in the presence of A3F. Single round infection assays were performed in the presence or absence of A3F as described in a legend to Fig. 5A. Values are presented as averages of more than 3 independent experiments. [file 1742-4690-6-1-S3.pdf]
